# Benchmarking of long-read assemblers for prokaryote whole genome sequencing

**DOI:** 10.12688/f1000research.21782.4

**Published:** 2021-02-01

**Authors:** Ryan R. Wick, Kathryn E. Holt

**Affiliations:** 1 Department of Infectious Diseases, Central Clinical School, Monash University, Melbourne, VIC, 3004, Australia; 2 Department of Infection Biology, London School of Hygiene & Tropical Medicine, London, WC1E 7HT, UK

**Keywords:** Assembly, long-read sequencing, Oxford Nanopore Technologies, Pacific Biosciences, microbial genomics, benchmarking

## Abstract

**Background: **Data sets from long-read sequencing platforms (Oxford Nanopore Technologies and Pacific Biosciences) allow for most prokaryote genomes to be completely assembled – one contig per chromosome or plasmid. However, the high per-read error rate of long-read sequencing necessitates different approaches to assembly than those used for short-read sequencing. Multiple assembly tools (assemblers) exist, which use a variety of algorithms for long-read assembly.

**Methods: **We used 500 simulated read sets and 120 real read sets to assess the performance of eight long-read assemblers (Canu, Flye, Miniasm/Minipolish, NECAT, NextDenovo/NextPolish, Raven, Redbean and Shasta) across a wide variety of genomes and read parameters. Assemblies were assessed on their structural accuracy/completeness, sequence identity, contig circularisation and computational resources used.

**Results: **Canu v2.1 produced reliable assemblies and was good with plasmids, but it performed poorly with circularisation and had the longest runtimes of all assemblers tested. Flye v2.8 was also reliable and made the smallest sequence errors, though it used the most RAM. Miniasm/Minipolish v0.3/v0.1.3 was the most likely to produce clean contig circularisation. NECAT v20200803 was reliable and good at circularisation but tended to make larger sequence errors. NextDenovo/NextPolish v2.3.1/v1.3.1 was reliable with chromosome assembly but bad with plasmid assembly. Raven v1.3.0 was reliable for chromosome assembly, though it did not perform well on small plasmids and had circularisation issues. Redbean v2.5 and Shasta v0.7.0 were computationally efficient but more likely to produce incomplete assemblies.

**Conclusions: **Of the assemblers tested, Flye, Miniasm/Minipolish, NextDenovo/NextPolish and Raven performed best overall. However, no single tool performed well on all metrics, highlighting the need for continued development on long-read assembly algorithms.

## Introduction

Genome assembly is the computational process of using shotgun whole-genome sequencing data (reads) to reconstruct an organism’s true genomic sequence to the greatest extent possible ^1^. Software tools which carry out assembly (assemblers) take sequencing reads as input and produce reconstructed contiguous pieces of the genome (contigs) as output.

If a genome contains repetitive sequences (repeats) which are longer than the sequencing reads, then the underlying genome cannot be fully reconstructed without additional information; i.e. if no read spans a repeat in the genome, then that repeat cannot be resolved, limiting contig length ^2^. Short-read sequencing platforms (e.g. those made by Illumina) produce reads hundreds of bases in length and tend to result in shorter contigs. In contrast, long-read platforms from Oxford Nanopore Technologies (ONT) and Pacific Biosciences (PacBio) can generate reads tens of thousands of bases in length which span more repeats and thus result in longer contigs ^3^.

Prokaryote genomes are simpler than eukaryote genomes in a few aspects relevant to assembly. First, they are smaller, most being less than 10 Mbp in size ^4^. Second, they contain less repetitive content and their longest repeat sequences are often less than 10 kbp in length ^5^. Third, prokaryote genomes are haploid and thus avoid assembly-related complications from diploidy/polyploidy ^6^. These facts make prokaryote genome assembly a more tractable problem than eukaryote genome assembly, and in most cases a long-read set of sufficient depth should contain enough information to generate a complete assembly – each replicon in the genome being fully assembled into a single contig ^7^. Prokaryote genomes also have two other features relevant to assembly: they may contain plasmids that differ from the chromosome in copy number and therefore read depth, and most prokaryote replicons are circular with no defined start/end point.

In this study, we examine the performance of various long-read assemblers in the context of prokaryote whole genomes. We assessed each tool on its ability to generate complete assemblies using both simulated and real read sets. We also investigated prokaryote-specific aspects of assembly, such as performance on plasmids and the circularisation of contigs.

## Methods

### Simulated read sets

Simulated read sets (read sequences generated *in silico* from reference genomes) offer some advantages over real read sets when assessing assemblers. They allow for a confident ground truth – i.e. the true underlying genome is known with certainty. They allow for large sample sizes, in practice limited only by computational resources. Also, a variety of genomes and read set parameters can be used to examine assembler performance over a wide range of scenarios. For this study, we simulated 500 read sets to test the assemblers, each using different parameters and a different prokaryote genome.

To select reference genomes for the simulated read sets, we first downloaded all bacterial and archaeal RefSeq genomes using ncbi-genome-download v0.2.10 (14333 genomes at the time of download) ^8^. We then performed some quality control steps: excluding genomes with a >10 Mbp chromosome, a <500 kbp chromosome, any >300 kbp plasmid, any plasmid >25% of the chromosome size or more than 9 plasmids ( *Extended data*, Figure S1) ^9^. We then ran Assembly Dereplicator v0.1.0 with a threshold of 0.1, resulting in 3153 unique genomes ^10^.

To produce a final set of 500 genomes with 500 plasmids, we randomly selected 250 genomes from those containing plasmids, repeating this selection until the genomes contained exactly 500 plasmids. We then added 250 genomes randomly selected from those without plasmids. Any ambiguous bases in the assemblies were replaced with ‘A’ to ensure that sequences contained only the four canonical DNA bases.

We then used Badread v0.1.5 to generate one read set for each input genome ^11^. The parameters for each set (controlling read depth, length, identity and errors) were randomly chosen to ensure a large amount of variability ( *Extended data*, Figure S2) ^9^. Note that not all of these read sets were sufficient to reconstruct the original genome (due to low depth or short read length), so even an ideal assembler would be incapable of completing an assembly for all 500 test sets.

For genomes containing plasmids, the read depth of plasmids relative to the chromosome was also set randomly, with limits based on the plasmid size ( *Extended data*, Figure S3) ^9^. Large plasmids were simulated at depths close to that of the chromosome while small plasmids spanned a wider range of depth. This was done to model the observed pattern that small plasmids often have a high per-cell copy number (i.e. may be high read depth) but can be biased against in library preparations (i.e. may be low read depth) ^12^. All replicons (chromosomes and plasmids) were treated as circular sequences in Badread, so the simulated read sets do not test assembler performance on linear sequences.

### Real read sets

Despite the advantages of simulated read sets, they can be unrealistic because read simulation tools (such as Badread) may not accurately model all relevant features: error profiles, read lengths, quality scores, etc. Real read sets are therefore also valuable when assessing assemblers. The challenge with real read sets is obtaining a ground truth genome against which assemblies can be checked. Since many reference genome sequences are produced using long-read assemblies, there is the risk of circular reasoning – if we use an assembly as our ground truth reference, our results will be biased in favour of whichever assembler produced the reference.

To avoid this issue, we used the datasets produced in a recent study comparing ONT (MinION R9.4) and PacBio (RSII CLR) data which also included Illumina reads for each isolate ^13^. For each of the 20 bacterial isolates in that study, we conducted two hybrid assemblies using Unicycler v0.4.7: Illumina+ONT and Illumina+PacBio ^14^. Unicycler works by first generating an assembly graph using the Illumina reads, then using long-read alignments to scaffold the graph’s contigs into a completed genome – a distinct approach from any of the long-read assemblers tested in this study. We ran the assemblies using Unicycler’s --no_miniasm option so it skipped its Miniasm-based step which could bias the results in favour of Miniasm/Minipolish. We then excluded any isolate where either hybrid assembly failed to reach completion or where there were >50 nucleotide differences between the two assemblies as determined by a Minimap2 alignment ^15^. I.e. the Illumina+ONT and Illumina+PacBio hybrid assemblies needed to be in near-perfect agreement with each other. This left six isolates for inclusion. The above process may have biased these isolates in favour of easier-to-assemble genomes, as more complex genomes would be more likely to encounter inconsistencies between the two Unicycler assemblies.

The ONT and PacBio read sets for these isolates were quite deep (156× to 535×) so to increase the number of assembly tests, we produced ten random read subsets of each, ranging from 40× to 100× read depth. This resulted in 120 total read sets for testing the assemblers (6 genomes × 2 platforms × 10 read subsets). The Illumina+ONT hybrid assembly was used as ground truth for each isolate.

All real and simulated read sets ^16^ and reference genomes ^17^ are available as *Underlying data*.

### Assemblers tested

We assembled each of the read sets using the current versions of eight long-read assemblers: Canu v2.1, Flye v2.8, Miniasm/Minipolish v0.3/v0.1.3, NECAT v20200803, NextDenovo/ NextPolish v2.3.1/v1.3.1, Raven v1.3.0, Redbean v2.5 and Shasta v0.7.0. Default parameters were used except where stated, and exact commands for each tool are given in the *Extended data*, Figure S4 ^9^. Assemblers that only work on PacBio reads (i.e. not on ONT reads) were excluded (HGAP ^18^, FALCON ^19^, HINGE ^20^ and Dazzler ^21^), as were hybrid assemblers which also require short read input (Unicycler ^14^ and MaSuRCA ^22^).

Canu has the longest history of all the assemblers tested, with its first release dating back to 2015. It performs assembly by first correcting reads, then trimming reads (removing adapters and breaking chimeras) and finally assembling reads into contigs ^23^. Its assembly strategy uses a modified version of the string graph algorithm ^24^, sometimes referred to as the overlap-layout-consensus (OLC) approach.

Flye takes a different approach to assembly: first combining reads into error-prone disjointigs, then collapsing repetitive sequences to make a repeat graph and finally resolving the graph’s repeats to make the final contigs ^25^. Of particular note to prokaryote assemblies, Flye has options for recovery of small plasmids ( --plasmids) and uneven depth of coverage ( --meta), both of which we used in this analysis.

Miniasm builds a string graph from a set of read overlaps – i.e. it performs only the layout step of OLC. It does not perform read overlapping which must be done separately with Minimap2, and it does not have a consensus step, so its assembly error rates are comparable to raw read error rates. A separate polishing tool such as Racon is therefore required to achieve high sequence identity ^26^. For this study, we developed a tool called Minipolish to simplify this process by conducting Racon polishing (two rounds by default) on a Miniasm assembly graph ^27^. To ensure clean circularisation of prokaryote replicons, circular contigs are ‘rotated’ (have their starting position adjusted) between polishing rounds. Minipolish also comes with a script ( miniasm_and_minipolish.sh) which carries out all assembly steps (Minimap2 overlapping, Miniasm assembly and Minipolish consensus) in a single command, and subsequent references to ‘Miniasm/Minipolish’ refer to this entire pipeline.

NECAT follows an approach similar to Canu: first correcting the input reads, then building an assembly from the corrected reads ^28^. Both the correction and assembly steps are progressive, using multiple processing steps to achieve better accuracy/completeness.

NextDenovo is a performance-oriented assembler, which like Canu and NECAT performs read-correction at the start of its pipeline. It performs the first two steps of OLC (overlap and layout), leaving the final step (consensus) to a separate tool: NextPolish ^29^. We used both tools in conjunction in this study, referred to as ‘NextDenovo/NextPolish’.

Raven (previously known as Ra) is another tool which takes an OLC approach to assembly ^30^. Its overlapping step shares algorithms with Minimap2, and its consensus step is based on Racon, making it similar to Miniasm/Minipolish. It differs in its layout step which includes novel approaches to remove spurious overlaps from the graph, helping to improve assembly contiguity.

Redbean (previously known as Wtdbg2) uses an approach to long-read assembly called a fuzzy Bruijn graph ^31^. This is modelled on the De Bruijn graph concept widely used for short-read assembly ^32^ but modified to work with the inexact sequence matches present in noisy long reads.

Shasta is an assembler designed for computational efficiency ^33^. To achieve this, much of its assembly pipeline is performed not directly on read sequences but rather on a reduced representation of marker *k*-mers. These markers are used to find overlaps and build an assembly graph from which a consensus sequence is derived.

### Computational environment

All assemblies were run on Ubuntu 18.04 instances of Australia’s Nectar Research Cloud which contained 32 vCPUs and 128 GB of RAM (r3.xxlarge flavour). To guard against performance variation caused by vCPU overcommit, the assemblers were limited to 16 threads (half the number of available vCPUs) in their options. Any assembly which exceeded 24 hours of runtime or 128 GB of memory usage was terminated.

### Assembly assessment

Our primary metric of assembly quality was contiguity, defined here as the longest single Minimap2 alignment between the assembly and the reference replicon, relative to the reference replicon length. This provides a simpler picture of assembly quality than is created by QUAST (which quantifies misassemblies and other metrics such as NG50) but is appropriate for cases where complete assembly is likely ^2^. Contiguity of exactly 100% indicates that the replicon was assembled completely with no missing or extra sequence ( *Extended data*, Figure S5A) ^9^. Contiguity of slightly less than 100% (e.g. 99.9%) indicates that the assembly was complete, but some bases were lost at the start/end of the contig ( *Extended data*, Figure S5B) ^9^. Contiguity of more than 100% (e.g. 101%) indicates that the contig contains duplicated sequence via start-end overlap ( *Extended data*, Figure S5C) ^9^. Much lower contiguity (e.g. 70%) indicates that the assembly was not complete due to fragmentation ( *Extended data*, Figure S5D) ^9^, missing sequence ( *Extended data*, Figure S5E) ^9^ or misassembly ( *Extended data*, Figure S5F) ^9^. Contiguity values were determined by aligning the contigs to a tripled version of the reference replicon, necessary to ensure that contigs can fully align even with start-end overlap and regardless of their starting position relative to that of the linearised reference sequence ( *Extended data*, Figure S6) ^9^. To encourage longer alignments, Minimap2 was run with the asm20 preset, chain elongation threshold of 10 kbp, banding threshold of 10 kbp, Z-drop score of 1000 and inversion Z-drop score of 500. The script for conducting this analysis (assess_assembly.py) is available in *Extended data*.

Contiguity values were determined for each replicon in the assemblies – e.g. if a genome contained two plasmids, then the assemblies of that genome have three contiguity values: one for the chromosome and one for each plasmid. A status of ‘fully complete’ was assigned to assemblies where all replicons (the chromosome and any plasmids if present) achieved a contiguity of *≥*99%. If an assembly had a chromosome with a contiguity of *≥*99% but incomplete plasmids, it was given a status of ‘complete chromosome’. If the chromosome had a contiguity of <99%, the assembly was deemed ‘incomplete’. If the assembly was empty or missing (possibly due to the assembler prematurely terminating with an error), it was given a status of ‘empty’. Computational metrics were also observed for each assembly: time to complete and maximum RAM usage.

## Results and discussion

**Figure 1.**
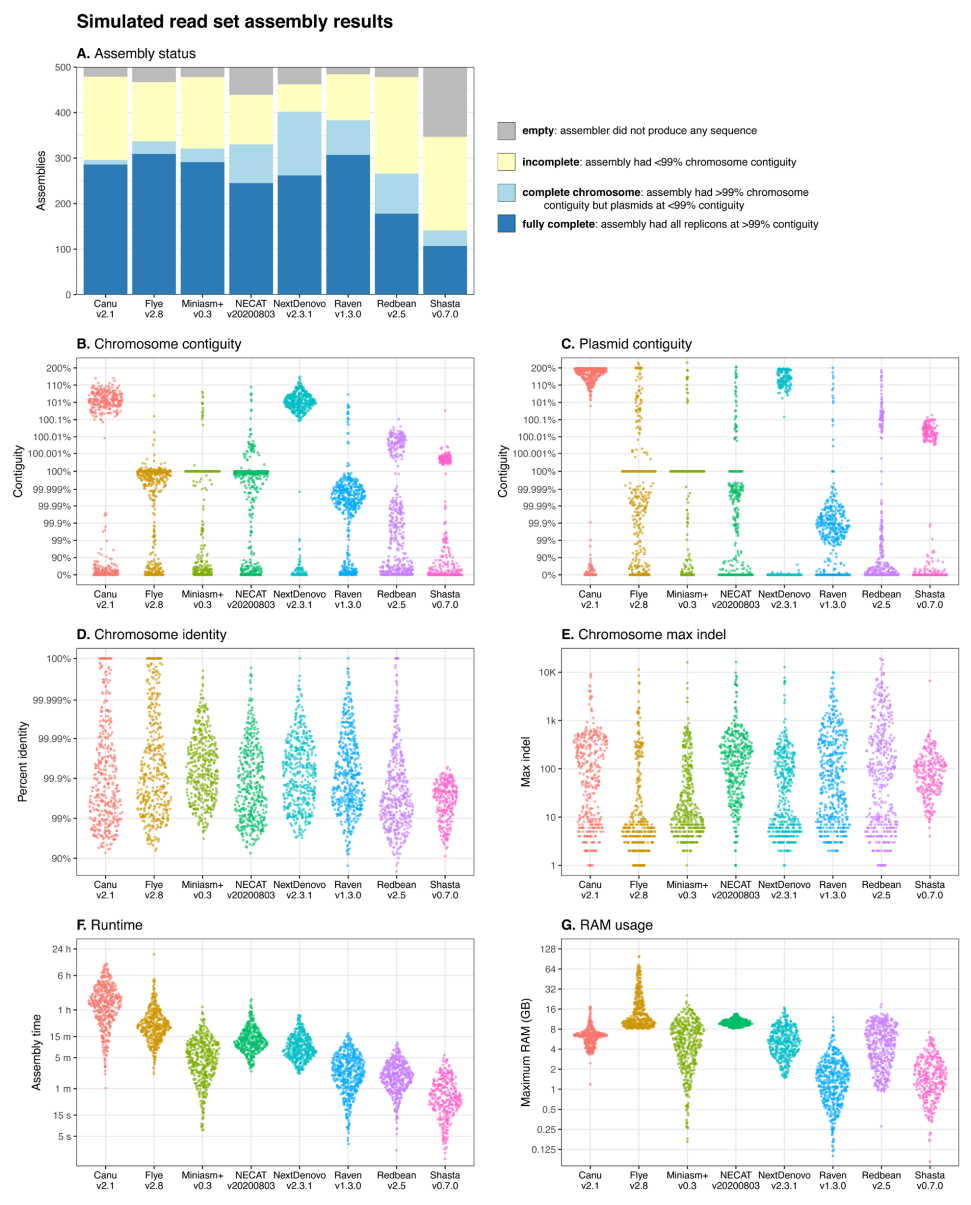
Assembly results for the simulated read sets, which cover a wide variety of parameters for length, depth and quality. ‘Miniasm+’ here refers to the entire Miniasm/Minipolish assembly pipeline. ( **A**) Proportion of each possible assembly outcome. ( **B**) Relative contiguity of the chromosome for each assembly, showing cleanliness of circularisation. ( **C**) Relative contiguity of all plasmids in the assemblies, showing cleanliness of circularisation. ( **D**) Sequence identity of each assembly’s longest alignment to the chromosome. ( **E**) The maximum indel error size in each assembly’s longest alignment to the chromosome. ( **F**) Total time taken (wall time) for each assembly. ( **G**) Maximum RAM usage for each assembly.

**Figure 2.**
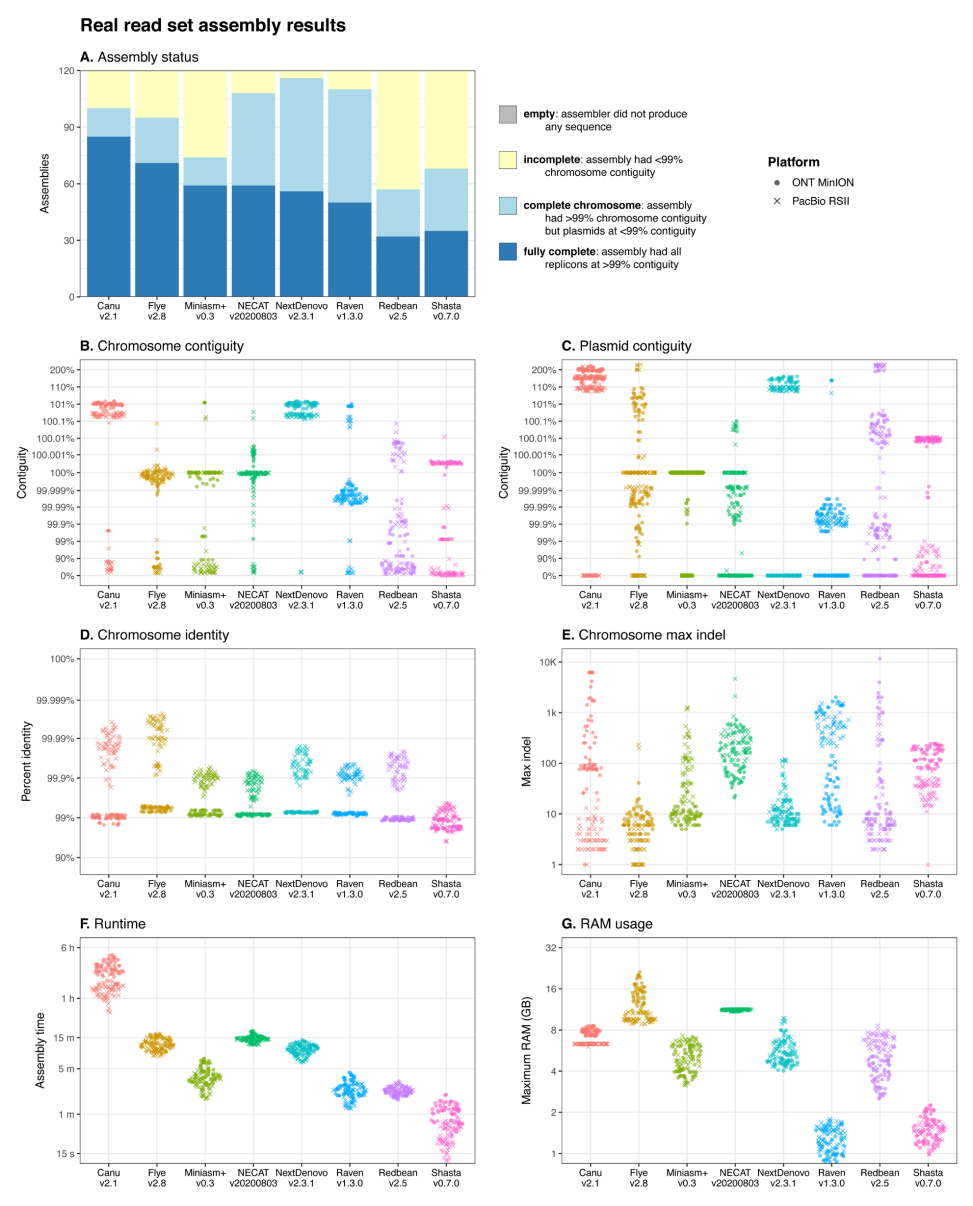
Assembly results for the real read sets, half containing ONT MinION reads (circles) and half PacBio RSII reads (X shapes). ‘Miniasm+’ here refers to the entire Miniasm/Minipolish assembly pipeline. ( **A**) Proportion of each possible assembly outcome. ( **B**) Relative contiguity of the chromosome for each assembly, showing cleanliness of circularisation. ( **C**) Relative contiguity of all plasmids in the assemblies, showing cleanliness of circularisation. ( **D**) Sequence identity of each assembly’s longest alignment to the chromosome. ( **E**) The maximum indel error size in each assembly’s longest alignment to the chromosome. ( **F**) Total time taken (wall time) for each assembly. ( **G**) Maximum RAM usage for each assembly.

Figure 1 and Figure 2 summarise the assembly results for the simulated and real read sets, respectively. Full tabulated results can be found in the *Extended data* ^9^. The assemblies, times and terminal outputs generated by each assembler are available as *Underlying data* ^34^. 

Figure 1A/ Figure 2A show the proportion of read sets with each assembly status. For the real read sets, a higher proportion of completed assemblies indicates a more reliable assembler – one which is likely to make a completed assembly given a typical set of input reads. For the simulated read sets, a higher proportion of completed assemblies indicates a more robust assembler – one which is able to tolerate a wide range of input read parameters, including adverse conditions such as low read accuracy and low read depth (conditions present in some of the simulated read sets but not in the real read sets). *Extended data*, Figure S7 ^9^ plots assembly contiguity against specific read set parameters to give a more detailed assessment of robustness. Plasmid assembly status, plotted with plasmid length and read depth, is shown in *Extended data*, Figure S8 and Figure S9 ^9^ for the simulated and real read sets, respectively.

Figure 1B/ Figure 2B show the chromosome contiguity values for each assembly, focusing on the range near 100%. These plots show how well assemblers can circularise contigs – i.e. whether sequence is duplicated or missing at the contig start/end ( *Extended data*, Figure S5) ^9^. The closer contiguity is to 100% the better, with exactly 100% indicating perfect circularisation. Plasmid contiguity values are shown in Figure 1C/ Figure 2C ^9^.

Assembly identity (consensus identity) is a measure of the base-level accuracy of an assembled contig relative to the reference sequence (how few substitution and small indel errors are present) and is shown in Figure 1D/ Figure 2D. The identity of assembled sequences is almost always higher than the identity of individual reads because errors can be ‘averaged out’ using read depth, producing more accurate consensus base calls. However, systematic read errors (e.g. mistakes in homopolymer length) can make perfect sequence identity difficult to achieve, regardless of assembly strategy ^35^. While most of the sequence inaccuracies are small (e.g. a single base indel), some can be much larger. Figure 1E/ Figure 2E show the size of the largest indel error found in each assembly’s chromosome, with smaller values being better. E.g. a maximum indel error size of 10 indicates that no indel errors larger than 10 bp were found in the chromosome.

Assembler resource usage is shown in terms of total runtime ( Figure 1F/ Figure 2F) and the maximum RAM usage during assembly ( Figure 1G/ Figure 2G).

### Reliability

Reliability was assessed using each assembler’s performance on the real read sets ( Figure 2A). When considering only the chromosome, NextDenovo/NextPolish was the most reliable assembler, followed by Raven, NECAT, Canu and Flye – all were able to complete the chromosome in over three-quarters of their assemblies. If plasmids are also considered, then Canu was the most reliable assembler followed by Flye. Miniasm/Minipolish and Shasta were moderately reliable, completing over half of the chromosomes. Redbean was the least reliable and completed less than half of the chromosomes.

### Robustness

Robustness was assessed using each assembler’s performance on the simulated read sets ( Figure 1A) which contained a large amount of variation on many metrics ( *Extended data*, Figure S7). NextDenovo/NextPolish and Raven were the most robust assemblers, able to complete the chromosome in over three-quarters of their assemblies. Flye, Redbean and Canu performed best in cases of low read depth, able to complete assemblies down to *~*10× depth ( *Extended data*, Figure S7A) ^9^. Raven, NextDenovo/NextPolish and NECAT performed best with low-identity read sets ( *Extended data*, Figure S7B) ^9^. The assemblers performed similarly with regards to read length, except for Shasta which required longer reads ( *Extended data*, Figure S7C) ^9^. The assemblers were similarly unaffected by random reads, junk reads, chimeric reads or adapter sequences ( *Extended data*, Figure S7D–F) ^9^. Read glitches (local breaks in continuity) were more likely to cause assembly problems for Canu, NECAT and Shasta ( *Extended data*, Figure S7G) ^9^.

### Identity

In our real read tests, Flye achieved the highest overall assembled sequence identity ( Figure 2D). Canu achieved high sequence identity on PacBio reads. Miniasm/Minipolish, NextDenovo/NextPolish and Raven did well on ONT reads. For each assembler, real PacBio reads resulted in higher identities than real ONT reads. For the simulated reads (which contain artificial error profiles), results were more erratic, with Canu and Flye performing best ( Figure 1D).

Regarding the maximum indel error size in the assemblies, Flye performed best, usually producing assemblies with errors no larger than 10 bp ( Figure 1E/ Figure 2E). NECAT and Shasta performed poorly, usually producing errors larger than 10 bp. The other assemblers had a large variance in this metric, sometimes producing assemblies with small errors and sometimes with large errors.

The nature of read errors depends on the sequencing platform and basecalling software used, so these results may not hold true for all read sets. Platform-specific post-assembly polishing tools (including Nanopolish ^7^, Medaka ^36^ and Arrow ^37^) are routinely used to improve the accuracy of long-read assemblies ^38^, and these can often achieve assembly identities of >99.9% for ONT read sets and >99.999% for PacBio read sets (i.e. better than any of the assemblers were able to achieve on their own). Identity can be further increased by polishing with Illumina reads where available (e.g. with Pilon ^39^). Therefore, the sequence identity produced by the assembler itself is potentially unimportant for many users. However, large-scale indel errors may be less easily fixed using polishing tools and therefore could be of greater relevance.

### Resource usage

Canu was the slowest assembler tested on both real ( Figure 2F) and simulated ( Figure 1F) read sets, sometimes taking hours to complete. Its runtime was correlated with read accuracy and read set size, with low-accuracy and large read sets being more likely to result in a long runtime.

Flye was typically faster than Canu, taking less than 15 minutes for the real read sets and usually less than an hour for the simulated read sets. It sometimes took multiple hours to assemble simulated read sets, and this was correlated with the amount of junk (low-complexity) reads, suggesting that removal of such reads via pre-assembly QC may be beneficial. Flye had the highest RAM usage of the tested assemblers and its RAM usage was correlated with read N50 and read set size, with long and large read sets being more likely to result in high RAM usage.

Shasta, Redbean and Raven were the fastest assemblers, typically completing assemblies in less than 5 minutes. While not tested in this study, Racon (which is used in Minipolish) and Raven can be run with GPU acceleration to further improve speed performance. Raven and Shasta had the lowest memory usage, typically requiring less than 4 GB of RAM.

### Circularisation

Of all assemblers tested, Miniasm/Minipolish and NECAT most regularly achieved exact circularisation (contiguity=100%) ( Figure 1B/ Figure 2B). Flye often excluded a small amount of sequence (tens of bases) from the start/end of circular contigs (contiguity <100%), and Raven typically excluded moderate amounts of sequence (hundreds of bases). Contiguities for Canu and NextDenovo/NextPolish usually exceeded 100%, indicating a large amount (thousands of bases) of start/end overlap. The amount of overlap in a Canu or NextDenovo/NextPolish assembly was correlated with the read N50 length ( *Extended data*, Figure S7C) ^9^. Redbean and Shasta were both erratic in their circularisation, often producing some sequence duplication (contiguity >100%) but occasionally dropping sequence (contiguity <100%).

In addition to cleanly circularising contig sequences, it is valuable for a prokaryote genome assembler to clearly distinguish between circular and linear contigs. This can provide users with a clue as to whether or not the genome was assembled to completion. Flye, Miniasm/Minipolish, Raven and Shasta produce graph files of their final assembly which can indicate circularity. Canu indicates circularity via the ‘suggestCircular’ text in its contig headers. NECAT, NextDenovo/NextPolish and Redbean do not signal to users whether a contig is circular.

### Plasmids

Canu and Flye were the two assemblers most able to assemble plasmids at a broad range of size and depth ( *Extended data*, Figures S8, S9) ^9^. Miniasm/Minipolish also performed well, though it failed to assemble plasmids if they were very small or had a very high read depth. Raven was able to assemble most large plasmids but not small plasmids. NECAT, NextDenovo/NextPolish, Redbean and Shasta were least successful at plasmid assembly.

Circularisation of plasmids followed the same pattern as for chromosomes, with Miniasm/Minipolish, Flye and NECAT most consistently achieving clean circularisation ( Figure 1C/ Figure 2C) ^9^. For smaller plasmids, start/end overlap could sometimes result in contiguities of *∼*200% – i.e. the plasmid sequence was duplicated in a single contig. This was most common with Canu and NextDenovo/NextPolish, though it occurred with other assemblers as well.

### Ease of use

Most assemblers tested were relatively easy to use, either running with a single command (Canu, Flye, Raven and Shasta) or providing a convenience script to bundle the commands together (Miniasm/Minipolish and Redbean). NECAT requires a configuration file be prepared, making it somewhat cumbersome to run. NextDenovo/NextPolish was the most difficult to run, requiring multiple commands and multiple configuration files. All were able to take long reads in FASTQ format as input ( *Extended data*, Figure S4) ^9^. We encountered no difficulty installing any of the tools by following the instructions provided.

Some of the assemblers needed a predicted genome size as input (Canu, NECAT, NextDenovo/NextPolish and Redbean) while others (Flye, Miniasm/Minipolish, Raven and Shasta) did not. This requirement could be a nuisance when assembling unknown isolates, as it may be hard to specify a genome size before the species is known.

### Configurability

While we ran our assemblies using default and/or recommended commands ( *Extended data*, Figure S4) ^9^, some of the assemblers have parameters which can be used to alter their behaviour. Raven was the least configurable assembler tested, with few options available to users. Flye offers some parameters, including overlap and coverage thresholds. Miniasm/Minipolish, NECAT, NextDenovo/NextPolish, Redbean and Shasta all offer more options, and Canu is the most configurable with hundreds of adjustable parameters. Many of the available parameters are arcane (e.g. Miniasm’s ‘max and min overlap drop ratio’ or Shasta’s ‘pruneIterationCount’), and only experienced power users are likely to adjust them – most will likely stick with default settings or only adjust easier-to-understand options. However, the presence of low-level parameters provides an opportunity to experiment and gain greater control over assemblies and are therefore appreciated even when unlikely to be used.

Another aspect worth noting is whether an assembler produces useful files other than its final assembly. Canu and NECAT stand out in this respect, as they create corrected and trimmed reads in their pipelines which have low error rates and are mostly free of adapters and chimeric sequences. Canu and NECAT can therefore be considered not just assemblers but also long-read correction tools suitable for use in other analyses.

### Assembler summaries

Canu v2.1 was the slowest assembler and suffered from large circularisation problems. However, it was quite reliable and did well with plasmids. Its main strength is in its configurability, so power users who are willing to learn Canu’s nuances may find that they can tune it to fit their needs. However, it is probably not the best choice for users wanting a quick and simple prokaryote genome assembly.

Flye v2.8 was a strong and well-balanced performer in our tests: reliable, robust and good with plasmids. It also produced the fewest large-scale indel errors in its assemblies. However, it often deleted some sequence (usually on the order of tens of bases) when circularising contigs and had the highest RAM usage of assemblers tested.

Miniasm/Minipolish v0.3/v0.1.3 was not the most reliable assembler but was fairly robust to read set parameters. Its main strength is that it was the most likely to consistently achieve perfect contig circularisation (as this is a specific goal of its polishing step). It was also one of the better assemblers for plasmids, especially regarding clean circularisation of plasmid sequences.

NECAT v20200803 performed reliably with chromosome assembly in the real read sets and was second only to Miniasm/Minipolish for contig circularisation. However, it failed to assemble many plasmids and was cumbersome to run.

NextDenovo/NextPolish v2.3.1/v1.3.1 was resource-efficient and very good at completing chromosomes in both simulated and real read sets, but it performed poorly on plasmid assembly. It was also the most cumbersome assembler to run, requiring multiple commands.

Raven v1.3.0 was reliable and robust for chromosome assembly and used very little RAM. However, it suffered from worse circularisation problems than Flye (often deleting hundreds of bases) and wasn’t good with small plasmids.

Redbean v2.5 assemblies tended to have glitches in the sequence which caused breaks in contiguity, making it perform poorly in both reliability and robustness. This makes it a less-than-ideal choice for long-read prokaryote read sets.

Shasta v0.7.0 was the fastest assembler tested and had low RAM usage, but it had the worst robustness and second-worst reliability. It is therefore more suited to assembly of large genomes in resource-limited settings (the use case for which it was designed) than it is for prokaryote genome assembly.

## Conclusions

Each of the different assemblers has pros and cons, and while no single assembler emerged as an ideal choice for prokaryote genome long-read assembly, the overall best performers were Flye, Miniasm/Minipolish, NextDenovo/NextPolish and Raven. Flye was reliable, especially for plasmid assembly, was the best performing assembler at low read depths and made the fewest large-scale sequence errors. Miniasm/Minipolish was the only assembler to consistently achieve clean contig circularisation. NextDenovo/NextPolish was best at generating complete chromosomal contigs. Raven was reliable for chromosome assembly, tolerant of low-identity read sets and computationally efficient.

For users looking to achieve an optimal assembly, we recommend trying multiple different tools and comparing the results. This will provide the opportunity for validation – confidence in an assembly is greater when it is in agreement with other independent assemblies. It also offers a chance to detect and repair circularisation issues, as different assemblers are likely to give different contig start/end positions for a circular replicon.

An ideal prokaryotic long-read assembler would reliably complete assemblies, be robust against read set problems, produce no large-scale errors, be easy to use, have low computational requirements, cleanly circularise contigs and assemble plasmids of any size. The importance of long-read assembly will continue to grow as long-read sequencing becomes more commonplace in microbial genomics, and so development of assemblers towards this ideal is crucial.

## Data availability

### Underlying data

Figshare: Read sets. https://doi.org/10.26180/5df6f5d06cf04 ^16^. 

These files contain the input read sets (both simulated and real) for assembly.

Figshare: Reference genomes. https://doi.org/10.26180/5df6e99ff3eed ^17^. 

This file contains the reference genomes against which the long-read assemblies were compared. For the simulated read sets, these genomes were the source sequence from which the reads were generated.

Figshare: Assemblies. https://doi.org/10.26180/5df6e2864a658 ^34^. 

These files contain assemblies (in FASTA format), times and terminal outputs for each of the assemblers.

### Extended data

Zenodo: Long-read-assembler-comparison. https://doi.org/10.5281/zenodo.2702442 ^9^.

This project contains the following extended data:

- Results (tables of results data, (including information on each reference genome, read set parameters and metrics foreach assembly).
- Scripts (scripts used to assess assemblies and generate plots).
- Figure S1. Distributions of chromosome sizes (A), plasmid sizes (B) and per-genome plasmid counts (C) for the reference genomes used to make the simulated read sets.
- Figure S2. Badread parameter histograms for the simulated read sets. (A) Mean read depths were sampled from a uniform distribution ranging from 5× to 200×. (B) mean read lengths were sampled from a uniform distribution ranging from 100 to 20000 bp. C: read length standard deviations were sampled from a uniform distribution ranging from 100 to twice that set’s mean length (up to 40000 bp). D: mean read identities were sampled from a uniform distribution ranging from 80% to 99%. (E) Max read identities were sampled from a uniform distribution ranging from that set’s mean identity plus 1% to 100%. (F) Read identity standard deviations were sampled from a uniform distribution ranging from 1% to the max identity minus the mean identity. (G, H and I) Junk, random and chimera rates were all sampled from an exponential distribution with a mean of 2%. (J) Glitch sizes/skips were sampled from a uniform distribution ranging from 0 to 100. (K) Glitch rates for each set were calculated from the size/skip according to this formula: 100000 */*1.6986 ^*s/*10^. (L) Adapter lengths were sampled from an exponential distribution with a mean of 50.
- Figure S3. Top: the target simulated depth of each replicon relative to the chromosome. The smaller the plasmid, the wider the range of possible depths. Bottom: the absolute read set of each replicon after read simulation.
- Figure S4. Commands used for each of the eight assemblers tested.
- Figure S5. Possible states for the assembly of a circular replicon. Reference sequences are shown in the inner circles in black and aligned contig sequences are shown in the outer circles in colour (red at the contig start to violet at the contig end). (A) Complete assembly with perfect circularisation. (B) Complete assembly but with missing bases leading to a gapped circularisation. (C) Complete assembly but with duplicated bases leading to overlapping circularisation. (D) Incomplete assembly due to fragmentation (multiple contigs per replicon). (E) Incomplete assembly due to missing sequence. (F) Incomplete assembly due to misassembly (noncontiguous sequence in the contig).
- Figure S6. Reference triplication for assembly assessment. (A) Due to the ambiguous starting position of a circular replicon, a completely-assembled contig will typically not align to the reference in a single unbroken alignment. (B) Doubling the reference sequence will allow for a single alignment, regardless of starting position. (C) However, if the contig contains start/end overlap (i.e. contiguity >100%) then even a doubled reference may not be sufficient to achieve a single alignment, depending on the starting position. (D) A tripled reference allows for an unbroken alignment, regardless of starting position, even in cases of >100% contiguity.
- Figure S7. Contiguity of the simulated read set assemblies plotted against Badread parameters for each of the tested assemblers. These plots show how well the assemblers tolerate different problems in the read sets. (A) Mean read depth (higher is better). (B) Max read identity (higher is better). (C) N50 read length (higher is better). (D) The sum of random read rate and junk read rate (lower is better). (E) Chimeric read rate (lower is better). (F) Adapter sequence length (lower is better). (G) Glitch size/skip (lower is better).
- Figure S8. Plasmid completion for the simulated read set assemblies for each of the tested assemblers, plotted with plasmid length and read depth. Solid dots indicate completely assembled plasmids (contiguity *≥*99%) while open dots indicate incomplete plasmids (contiguity <99%). Percentages in the plot titles give the proportion of plasmids which were completely assembled.
- Figure S9. Plasmid completion for the real read set assemblies for each of the tested assemblers, plotted with plasmid length and read depth. Solid dots indicate completely assembled plasmids (contiguity *≥*99%) while open dots indicate incomplete plasmids (contiguity <99%). Percentages in the plot titles give the proportion of plasmids which were completely assembled.

Extended data are also available on GitHub.

Data are available under the terms of the Creative Commons Attribution 4.0 International license (CC-BY 4.0).
