# Supplementary material for: Benchmarking of long-read assemblers for prokaryote whole genome sequencing
Source: F1000Res. 2021 Feb 1;8:2138. Originally published 2019 Dec 23. [Version 4] doi: 10.12688/f1000research.21782.4 (PMC6966772; doi:10.12688/f1000research.21782.4)
Supplement: Peer review history

# Peer review: Reviewer response for version 1

**DOI:** 10.5256/f1000research.24010.r58116

**Published:** 2020-01-30

**Authors:** Olin Silander

**Affiliations:** 1 School of Natural and Computational Sciences, Massey University Auckland, North Shore, New Zealand

The authors compare six long read genome assemblers using simulated and real data (PacBio and Nanopore). They find that there is no single best method, and that each offers distinct advantages and disadvantages.

 I enjoyed reading this paper. It was well written and clearly presented. As I understand, the authors plan to continually update the benchmarking is a fantastic step forward and considerably improves the utility of such a publication. This should be noted more explicitly in the manuscript.

**Major comments:**

- P.3 “Real Read Sets”. Could the authors note which fraction of the PacBio reads were CCS / HiFi reads?
- p.4 para.1: We then excluded any isolate where either hybrid assembly failed to reach completion or where there were structural differences between the two assemblies as determined by a Minimap2 alignment.
  I wonder if this biases the genomes that were used such that they were easier to assemble than the genomes that were left out. I do not have a big problem with this, but it could be mentioned. It would also be good to provide slightly more detail on what precisely “structural differences between the two assemblies” means - e.g. does this include large indels (size range), inversions, etc.
- P.5 para.4: Figure 1B/Figure 2B shows the chromosome contiguity values for each assembly.
  There are some interesting patterns in 1B and 2B. First is the large number of Shasta assemblies have precisely 100.005% contiguity (looks to be mostly ONT assemblies). I am also surprised by the sort of bimodality in 1C/2C flye assemblies (and somewhat the miniasm assemblies). I would expect an even spread, but instead it looks like some assemblies have similar to 99% identity, whereas others have ~ 2-fold lower error rate (99.5% identity, my guesstimate). Is there an explanation for either of these patterns?
- P.5 Discussion of Identity. The authors could note the level generally achieved by polishing, which for ONT I think is around 99.98% (I am sure the authors are more aware than I am).

Is the work clearly and accurately presented and does it cite the current literature?

Yes

If applicable, is the statistical analysis and its interpretation appropriate?

Yes

Are all the source data underlying the results available to ensure full reproducibility?

Yes

Is the study design appropriate and is the work technically sound?

Yes

Are the conclusions drawn adequately supported by the results?

Yes

Are sufficient details of methods and analysis provided to allow replication by others?

Yes

Reviewer Expertise:

Microbial genomics and evolution, transcription, metagenomics

I confirm that I have read this submission and believe that I have an appropriate level of expertise to confirm that it is of an acceptable scientific standard.

# Response

**Published:** 2020-04-15

**Authors:** Ryan Wick

**Affiliations:** Monash University, Australia

We thank the reviewer for their feedback, and changes to the article will be incorporated in its next version (along with updated results for newer assemblers/versions).

**Regarding point number 1:**

 None of the PacBio read sets were CCS – all were CLR. We have clarified this in the main text of the paper, noting that they are CLR reads when first introduced.

**Regarding point number 2:**

 We have clarified both of these points in the text. The relevant section now reads: ‘We then excluded any isolate where either hybrid assembly failed to reach completion or where there were >50 nucleotide differences between the two assemblies as determined by a Minimap2 alignment. I.e. the Illumina+ONT and Illumina+PacBio hybrid assemblies needed to be in near-perfect agreement with each other. This left six isolates for inclusion. The above process may have biased these isolates in favour of easier-to-assemble genomes, as more complex genomes would be more likely to encounter inconsistencies between the two Unicycler assemblies.’

**Regarding point number 3:**

 These are indeed interesting patterns, but I can only speculate as to what the explanations are. Shasta is prone to producing ~10-15 bp of overlap in its assemblies. This may be related to the fact that Shasta operates on a reduced representation of the read sequences that is based on 10-mers. The bimodality of the Flye ONT assembly identity distribution is not as pronounced for the newer version of Flye (v2.7) but it is still there. The identity is relatively consistent within each genome (e.g. two read sets for a given genome tend to yield similar assembly identity), so I would speculate that the cause has something to do with the genome itself. E.g. perhaps the lower identity genomes have some type of DNA modification motif that is more likely to cause errors in the consensus sequence.

**Regarding point number 4:**

 We have added to the text to elaborate on polished assembly identity: ‘Platform-specific post-assembly polishing tools (including Nanopolish, Medaka and Arrow) are routinely used to improve the accuracy of long-read assemblies, and these can often achieve assembly identities of >99.9% for ONT read sets and >99.999% for PacBio read sets (i.e. better than any of the assemblers were able to achieve on their own).’

# Peer review: Reviewer response for version 1

**DOI:** 10.5256/f1000research.24010.r58301

**Published:** 2020-01-22

**Authors:** Mikhail Kolmogorov

**Affiliations:** 1 Department of Computer Science and Engineering, University of California San Diego, La Jolla, USA

The article presents the benchmarking of the current popular long-read assemblers (Canu, Flye, Miniasm/Minipolish, Raven, Redbean and Shasta) on various prokaryotic genomes. Wick & Holt have simulated 500 long-read datasets to reflect various genomic features (such as repeat length and complexity) as well as different sequencing parameters (depth, read length, sequencing artifacts etc). In addition, the authors test the assemblers on 160 real PacBio and Oxford Nanopore datasets. For each benchmarked algorithm, Wick & Holt summarize the important assembly metrics, such as contiguity or base-level accuracy (measured against the corresponding references), as well as overall user experience.

 The manuscript is well-written, and the study design is sound. The presented benchmarks will be a valuable resource for the long-read genomics community, both for developers and users. Importantly, the authors have made all data sets and benchmarking pipelines freely available. I only have the following minor suggestions:

1. In my view, the evaluation pipeline designed by the authors could be highlighted more in the main text. E.g. how can a developer test a different assembler using the described benchmarks? Is it quick to reproduce? What would be the resource requirements?
2. It would be useful to compare the pros and cons of this work with the other assembly evaluation methods (such as QUAST) in a short discussion.
3. On Figure 2, triangles and circles are somewhat difficult to distinguish. Is there a way to better visually separate PacBio and ONT data points (maybe color tones or background pattern)?
4. For the sake of completeness, it is worth mentioning the minimap2 alignment identity threshold that is used for contiguity evaluation.
5. DOI links to read sets and generated assemblies seem to have an unneeded space that break the URLs.

Is the work clearly and accurately presented and does it cite the current literature?

Yes

If applicable, is the statistical analysis and its interpretation appropriate?

Not applicable

Are all the source data underlying the results available to ensure full reproducibility?

Yes

Is the study design appropriate and is the work technically sound?

Yes

Are the conclusions drawn adequately supported by the results?

Yes

Are sufficient details of methods and analysis provided to allow replication by others?

Yes

Reviewer Expertise:

Bioinformatics, genomics

I confirm that I have read this submission and believe that I have an appropriate level of expertise to confirm that it is of an acceptable scientific standard.

# Response

**Published:** 2020-04-15

**Authors:** Ryan Wick

**Affiliations:** Monash University, Australia

We thank the reviewer for their feedback, and changes to the article will be incorporated in its next version (along with updated results for newer assemblers/versions).

**Regarding point number 1:**

We have refined the script used to assess assemblies to make it more generalisable and usable: command line help text and usage information at the top of the script. We have also added a mention of the script and where it can be found to the main text of the paper: ‘The script for conducting this analysis (assess_assembly.py) is available in *Extended data*.’

**Regarding point number 2:**

We have added a brief comparison between our evaluation metric (contiguity) and QUAST to the main text: ‘This provides a simpler picture of assembly quality than is created by QUAST (which quantifies misassemblies and other metrics such as NG50) but is appropriate for cases where complete assembly is likely.’

**Regarding point number 3:**

We have changed the triangles for PacBio data points to X shapes, which are easier to distinguish from the circles used for ONT data points.

**Regarding point number 4:**

We have added the exact minimap2 options used to the main text of the article: ‘To encourage longer alignments, Minimap2 was run with the asm20 preset and chain elongation and banding thresholds of 10 kbp.’

**Regarding point number 5:**

We have removed the space to fix the links for these URLs.​​​​​​​

# Peer review: Reviewer response for version 1

**DOI:** 10.5256/f1000research.24010.r58113

**Published:** 2020-01-16

**Authors:** Mile Šikić, Robert Vaser

**Affiliations:** 1 Department of Electronic Systems and Information Processing, Faculty of Electrical Engineering and Computing, University of Zagreb, Zagreb, Croatia; 2 Faculty of Electrical Engineering and Computing, University of Zagreb, Zagreb, Croatia; 3 Genome Institute of Singapore, A*STAR, Singapore

The authors present a benchmark regarding prokaryotic organisms for several state-of-the-art long-read assemblers. The comparison includes both third generation sequencing technologies with real and simulated data, assessing various assembly traits with the conclusion that no assembler is perfect. The manuscript is well written, the figures look neat and all the data is freely available online.

 Minor comments:

1. Generating the assembly with a hybrid approach which is different from all benchmarked assemblers is a good approach, but is there a possibility to analyse in details datasets which have reference genomes assembled with Sanger sequencing (such as CFT073 and MGH78578 datasets used in **De Maio N, Shaw LP, Hubbard A, *et al.*** ^1^)?
2. As minipolish is a new pipeline introduced in this paper, I would suggest describing it a bit more in detail.
3. Ra assembler has been published as a conference proceedings here.

Is the work clearly and accurately presented and does it cite the current literature?

Yes

If applicable, is the statistical analysis and its interpretation appropriate?

Not applicable

Are all the source data underlying the results available to ensure full reproducibility?

Yes

Is the study design appropriate and is the work technically sound?

Yes

Are the conclusions drawn adequately supported by the results?

Yes

Are sufficient details of methods and analysis provided to allow replication by others?

Yes

Reviewer Expertise:

Sequence alignment, de novo assembly, algorithms, machine learning

We confirm that we have read this submission and believe that we have an appropriate level of expertise to confirm that it is of an acceptable scientific standard.

## References

[1] : Comparison of long-read sequencing technologies in the hybrid assembly of complex bacterial genomes. *Microb Genom*.2019;5(9) : 10.1099/mgen.0.000294 10.1099/mgen.0.000294 31483244PMC6807382

# Response

**Published:** 2020-04-15

**Authors:** Ryan Wick

**Affiliations:** Monash University, Australia

We thank the reviewer for their feedback, and changes to the article will be incorporated in its next version (along with updated results for newer assemblers/versions).

**Regarding point number 1:**

 We were reluctant to use Sanger-finished genomes as references for this study due to the dynamic nature of bacterial genomes. I.e. when a strain is sequenced multiple times from separate colonies and DNA extractions, there can be discrepancies between the underlying genomes. We encountered this problem when benchmarking Unicycler using public datasets for the *E. coli* K-12 MG1655 genome ( 10.1371/journal.pcbi.1005595). In that case, an insertion sequence had shifted in the genome relative to the Sanger-finished reference, causing false positive misassemblies. Scenarios such as this would be detrimental in our current study where even a single such discrepancy could seriously impact the contiguity metric we used (which requires zero misassemblies to achieve a contiguity of 100%). Instead, we opted to produce our own reference sequences (as described in the article) using De Maio et al’s single DNA extraction per isolate.

**Regarding point number 2:**

 Further information on the Minipolish process is available on its GitHub page. We have now created a DOI for this repository to make a permanent digital record ( 10.5281/zenodo.3752203) and added it to the article’s references.

**Regarding point number 3:**

 We have updated the article’s reference for Ra to the provided conference proceedings.

# Peer review: Reviewer response for version 1

**DOI:** 10.5256/f1000research.24010.r58115

**Published:** 2020-01-09

**Authors:** Steven L Salzberg, Aleksey Zimin

**Affiliations:** 1 Department of Biomedical Engineering, Johns Hopkins University, Baltimore, Maryland, USA; 2 Department of Biomedical Engineering, Johns Hopkins University School of Medicine, Baltimore, Maryland, USA; 3 Department of Computer Science, Whiting School of Engineering, Johns Hopkins University, Baltimore, Maryland, USA; 4 Department of Biostatistics, Bloomberg School of Public Health, Johns Hopkins University, Baltimore, Maryland, USA

The report is clear and concise, easy to read, and the authors' conclusions are well supported by their experimental results. The authors are to be commended for their unusual attention to reproducibility, and for making all data easily available.

 We just have a couple of minor suggestions:

1. Reliability vs. robustness: the authors summarized their findings using the terms "reliability" for performance on real data sets, and "robustness" on simulated data sets. These terms might be a bit misleading to some readers. Reliability can be defined as consistent performance with good results, and robustness (in contrast) might be the ability to perform well under adverse conditions. The real data sets do vary in quality and coverage, although not as much as the simulated data. But it seems that both reliability and robustness can be evaluated on both types of data. If they want to use the term "robustness," perhaps they could also plot the number of successful assemblies (or contiguity) vs the read error rate for each assembler. In this respect, a high error rate might be considered an adverse condition.
2. Figure 1 is excellent, and provides a really nice summary of the performance on simulated data. However, only 1 of the programs, Flye, failed due to running out of memory, which was limited to 64 GB of RAM. Flye was otherwise one of the best performers. RAM is fairly inexpensive today, and it's not hard to find a server with >64 GB. The Figure doesn't show how much more memory Flye would need, and it would be really helpful to know that. Would 128GB allow it to complete in all cases? We suggest they run those failed assemblies on a larger-memory server and report what was needed.
  Another consideration here, though, is that depending on overcommit ratio and swap parameters, processes may be killed or slowed down long before they reach the 64GB physical memory limit. The impact of swap space on performance is an unknown here as well. For a clean evaluation, they should be sure (and maybe they did this, we can't tell) that swap was disabled and that the overcommit ratio was set to 97% to allow a process to use essentially all avaliable RAM.  (There's more information about memory overcommit settings here) If swapping came into play on any of these jobs, then it would drastically increase runtime.

Is the work clearly and accurately presented and does it cite the current literature?

Yes

If applicable, is the statistical analysis and its interpretation appropriate?

Not applicable

Are all the source data underlying the results available to ensure full reproducibility?

Yes

Is the study design appropriate and is the work technically sound?

Yes

Are the conclusions drawn adequately supported by the results?

Yes

Are sufficient details of methods and analysis provided to allow replication by others?

Yes

Reviewer Expertise:

Genomics, computational biology

We confirm that we have read this submission and believe that we have an appropriate level of expertise to confirm that it is of an acceptable scientific standard.

# Response

**Published:** 2020-04-15

**Authors:** Ryan Wick

**Affiliations:** Monash University, Australia

We thank the reviewer for their feedback, and changes to the article will be incorporated in its next version (along with updated results for newer assemblers/versions).

**Regarding point number 1:**

 Supplementary figure S7 (available here) plots assembly contiguity against many different parameters used to generate the simulated reads, including maximum read identity. This gives a more detailed look at assembler ‘robustness’ towards a number of adverse conditions. Also, in the main text where the terms ‘reliability’ and ‘robustness’ are introduced, we have clarified that the simulated read sets contain adverse conditions which are not present in the real read sets.

**Regarding point number 2:**

 We have created a new virtual machine on the Nectar Research Cloud with 128 GB of RAM (the most available in that service) and all new results (including those for Flye v2.7) were run on this VM. This has prevented assemblies from failing due to lack of memory. Since the larger VM allowed all assemblies to complete, we have opted to not alter the Linux memory settings and instead use the defaults. We checked memory statistics (as reported by /usr/bin/env time -v) and saw that major page fault counts were low (usually zero, sometimes in the tens and occasionally a few hundred for Canu), so we don’t believe that memory swapping has significantly impacted performance.
